# Hydroethanolic *Montanoa bipinnatifida* Leaf Extract Attenuates Acute Ethanol-Induced Gastric Mucosal Injury in Female CD-1 Mice: Associations with Lipid Peroxidation and IL-1β Expression

**DOI:** 10.3390/nu18193139

**Published:** 2026-09-23

**Authors:** Verónica Edith Gallegos-Hernández, Ehekatzin García-Valdés, Margarita Gómez-Chavarín, Maria-del-Carmen Silva-Lucero, Jorge Saldaña-Mancilla, Ruth Bustamante-García, Atonatiu E. Gómez-Martínez, Ana María Vázquez-Alvarez, Mónica Rosalía Jaime-Fonseca, Patricia Vergara-Aragón

**Affiliations:** 1 Centro de Investigación en Ciencia Aplicada y Tecnología Avanzada (CICATA), Instituto Politécnico Nacional, Av. Legaria 694, Col. Irrigación, Miguel Hidalgo, Ciudad de México C.P. 11500, Mexico; gavh940112@gmail.com (V.E.G.-H.); 09ehekatzin94@gmail.com (E.G.-V.); 2 Laboratorio de Biotecnología, Departamento de Fisiología, Facultad de Medicina, Universidad Nacional Autónoma de México (UNAM), Av. Universidad 3000, Coyoacán, Ciudad de México C.P. 04510, Mexico; margaritachavarin@gmail.com (M.G.-C.); carmenaguila10@hotmail.com (M.-d.-C.S.-L.); jorgesaldanamancilla@gmail.com (J.S.-M.); 3 Departamento de Biología, Facultad de Química, Universidad Nacional Autónoma de México (UNAM), Av. Universidad 3000, Coyoacán, Ciudad de México C.P. 04510, Mexico; fruthb@gmail.com (R.B.-G.); anavazquez@quimica.unam.mx (A.M.V.-A.)

**Keywords:** *Montanoa bipinnatifida*, gastroprotection, ethanol-induced gastric injury, oxidative stress, lipid peroxidation, polyphenols, IL-1β

## Abstract

**Background/Objectives:** Ethanol-induced gastric injury is a widely used experimental model for evaluating gastroprotective interventions because it reproduces key features of gastric mucosal damage, including oxidative stress, lipid peroxidation, inflammation, and epithelial disruption. Previous phytochemical characterization of *Montanoa bipinnatifida* leaves revealed a rich profile of bioactive polyphenols with antioxidant potential. Therefore, this study investigated the gastroprotective effects of a hydroethanolic leaf extract of *M. bipinnatifida* in female CD-1 mice subjected to ethanol-induced gastric injury. **Methods:** Animals received the extract orally for seven consecutive days before administration of 50% ethanol. Gastric tissues were subsequently evaluated by macroscopic, histopathological, biochemical, and phytochemical analyses. **Results:** Ethanol exposure induced severe gastric damage characterized by hemorrhagic lesions, epithelial degeneration, mucosal edema, and inflammatory cell in-filtration. In contrast, pretreatment with the extract significantly reduced gastric injury, preserving tissue architecture, and attenuating histopathological alterations. Biochemical and molecular analyses revealed a marked decrease in malondialdehyde levels and IL-1β expression following extract pretreatment, whereas TNF-α expression was not significantly reduced; SOD2 and catalase protein expression showed no significant treatment-associated changes. HPLC-DAD analysis confirmed the presence of catechin and procyanidin B2 in the extract. **Conclusions:** Overall, these findings indicate that *M. bipinnatifida* leaf extract attenuates acute ethanol-induced gastric injury and that this gastroprotective response is associated with reduced lipid peroxidation and selective attenuation of IL-1β rather than generalized suppression of the inflammatory markers examined. These results support further investigation of *M. bipinnatifida* as a potential source of gastroprotective bioactive compounds.

## 1. Introduction

Gastric mucosal injury is a multifactorial pathological process associated with alcohol consumption, nonsteroidal anti-inflammatory drugs, *Helicobacter pylori* infection, stress, and impaired gastric microcirculation. Gastric homeostasis depends on the balance between aggressive factors and endogenous protective mechanisms, including mucus and bicarbonate secretion, epithelial restitution, prostaglandin synthesis, mucosal blood flow, and antioxidant protection. Disruption of this balance results in epithelial damage, inflammatory cell infiltration, and ulcerative lesions [1,2,3]. Among experimental models, ethanol-induced gastric injury is widely used to evaluate gastroprotective agents because it reproduces the main features of acute gastric mucosal damage. Ethanol rapidly penetrates the gastric mucosa, disrupts membrane integrity, impairs mitochondrial function, and increases vascular permeability, leading to epithelial exfoliation, edema, hemorrhage, and necrosis. These alterations are primarily driven by excessive reactive oxygen species (ROS) generation, which promotes lipid peroxidation, oxidative stress, activation of pro-inflammatory signaling pathways, and depletion of endogenous antioxidant defenses, ultimately exacerbating mucosal injury [4]. Oxidative stress plays a central role in gastric mucosal injury by overwhelming endogenous antioxidant defenses and promoting lipid peroxidation, cellular dysfunction, and inflammation. Increased malondialdehyde (MDA) levels, depletion of reduced glutathione (GSH), and elevated myeloperoxidase (MPO) activity reflect redox imbalance and neutrophil infiltration during gastric damage. These processes are regulated by signaling pathways such as NF-κB and Nrf2, which modulate inflammatory and antioxidant responses. Therefore, targeting the redox–inflammatory axis represents a key strategy for gastric mucosal protection [5,6,7]. In this context, plant-derived bioactive compounds, particularly polyphenols, have received increasing attention as sources of gastroprotective agents. Polyphenol-rich medicinal plants are particularly relevant due to their ability to modulate oxidative stress and inflammatory signaling pathways. Compounds such as catechin and procyanidin B2 have demonstrated antioxidant, anti-inflammatory, and cytoprotective effects in experimental models of gastrointestinal and tissue injury [8,9,10].

*Montanoa bipinnatifida* C. Koch, commonly known as “Penumbra” is a medicinal plant of ethnopharmacological relevance in Mexico. Although several species of the genus *Montanoa* have demonstrated antioxidant and anti-inflammatory properties attributable to their polyphenolic constituents, the gastroprotective activity of *M. bipinnatifida* has not yet been experimentally characterized [11,12]. This represents a significant gap in knowledge, particularly considering that members of the Asteraceae family are rich in polyphenols, flavonoids, and sesquiterpene lactones, compounds widely associated with gastroprotective mechanisms such as enhancement of mucus production, modulation of prostaglandin pathways, and attenuation of oxidative stress [13,14]. In this context, plant-derived bioactive compounds, particularly polyphenols, have received increasing attention as sources of gastroprotective agents. Polyphenol-rich medicinal plants are particularly relevant due to their ability to modulate oxidative stress and inflammatory signaling pathways. Recent studies have further supported the gastroprotective potential of Asteraceae-derived plants and their bioactive constituents, for example, eupatilin, a polymethoxyflavone isolated from *Artemisia argyi*, attenuated ethanol-induced gastric mucosal injury through effects associated with oxidative stress and NF-κB signaling [15,16]. Similarly, *Achillea erbarotta* subsp. *moschata* has shown gastrointestinal and anti-inflammatory activities in preclinical studies [17]. Despite its traditional use, no experimental evidence currently demonstrates whether *M. bipinnatifida* protects gastric tissue against oxidative gastric injury or identifies the phytochemical constituents potentially associated with this activity. Therefore, the present study aimed to evaluate the gastroprotective effects of a hydroethanolic leaf extract of *M. bipinnatifida* in a mouse model of acute ethanol-induced gastric mucosal injury and to examine their association with oxidative stress, lipid peroxidation, and selected inflammatory and antioxidant-related molecular responses. We hypothesized that oral administration of the extract would attenuate ethanol-induced gastric mucosal injury and that this effect would be associated with reduced lipid peroxidation and modulation of inflammatory and antioxidant-related responses. Accordingly, the main research questions were: (1) whether the extract produces dose-dependent protection against ethanol-induced gastric injury; (2) whether the selected effective dose reduces gastric lipid peroxidation, as assessed by malondialdehyde (MDA) levels; and (3) whether gastroprotection is accompanied by changes in selected molecular markers, particularly IL-1β, TNF-α, SOD2, and catalase. In addition, the phytochemical profile of the extract was qualitatively characterized by HPLC-DAD to identify major phenolic constituents potentially associated with the observed effects.

## 2. Materials and Methods

### 2.1. Plant Material and Botanical Identification

Leaves of *Montanoa bipinnatifida* C. Koch (Asteraceae), locally known as “Penumbra”, were collected in September 2022 from the municipality of Berriozábal, located within the metropolitan region of Tuxtla Gutiérrez, Chiapas, Mexico (16°47′ N, 93°16′ W; approximately 820 m above sea level). Botanical authentication was performed through detailed morphological comparison with taxonomically validated specimens deposited at the National Herbarium of Mexico (MEXU), Institute of Biology, National Autonomous University of Mexico (UNAM). A voucher specimen (MB-2022-01) was deposited in the institutional herbarium to ensure traceability and future reference.

### 2.2. Preparation of the Hydroethanolic Extract and Phytochemical and Chromatographic Characterization

The hydroethanolic extract of *M. bipinnatifida* (Pen) leaves was prepared according to the procedure previously described by Gallegos-Hernández et al. [18]. Briefly, air-dried leaves (300 gr) were finely ground in a disk mill (Model 148-2, The Bauer Co., Worthington, PA, USA), sieved (300 μm), and subjected to extraction via maceration with 70% ethanol (1:10, *w*/*v*) at room temperature (22 ± 2 °C) in the absence of light for 15 days. After extraction, the solvent was re-moved under reduced pressure at 45 ± 2 °C using a rotary evaporator (RE-500, Yamato, Tokyo, Japan), the residual solvent was removed at room temperature (22 ± 2 °C) and the resulting crude extract was stored at 4 °C in amber containers until use. The resulting crude hydroethanolic extract showed an extraction yield of 18.18%, calculated relative to the initial dry plant material. A comprehensive phytochemical characterization of the extract had been previously established through complementary analytical approaches including UV–visible spectroscopy, Fourier-transform infrared spectroscopy (FTIR), electrospray ionization Fourier transform ion cyclotron resonance mass spectrometry (ESI-FT-ICR-MS), gas chromatography–mass spectrometry (GC–MS), and in vitro antioxidant assays [16]. The present study did not repeat the full characterization; instead, HPLC.DAD was performed to verify the phenolic profile of the batch used in the animal experiments. These analyses revealed a chemically complex matrix enriched in phenolic acids, flavan-3-ols, proanthocyanidins, and other redox-active phytoconstituents with recognized antioxidant and anti-inflammatory potential. Given the central role of oxidative stress in ethanol-induced gastric injury, additional chromatographic analyses were performed in the present study to verify the presence of major phenolic compounds potentially involved in the gastroprotective activity of the ex-tract.

### 2.3. HPLC-DAD Analysis of Major Phenolic Compounds

High-performance liquid chromatography coupled with diode-array detection (HPLC-DAD) was employed to identify major phenolic compounds within the hydroethanolic extract following validated analytical procedures widely used for the characterization of plant-derived polyphenols [19]. The extract was dissolved in methanol (LC-MS grade, Sigma-Aldrich, St. Louis, MO, USA) at a 0.1 mg/mL concentration, sonicated for 5 min, and filtered through a 0.22 μm hydro-philic PVDF membrane filter (Millipore, NA, USA). Chromatographic separation was carried out using an Agilent 1200 HPLC system equipped with a Zorbax Eclipse Plus C18 column (100 × 4.6 mm, 3.5 μm) (Agilent Technologies, Santa Clara, CA, USA). The mobile phase consisted of (A) ultrapure water containing 0.1% formic acid and (B) methanol/acetonitrile (50:50, *v*/*v*). Elution was performed using the following gradient profile: 10–15% B (0–10 min), 15–16% B (10–15 min), 16–20% B (15–20 min), and 20–35% B (20–30 min). The flow rate was maintained at 0.7 mL min^−1^, the column temperature at 30 °C, and the injection volume at 5 μL. Detection was monitored at 270 nm, a wavelength particularly suitable for flavan-3-ols and condensed tannins. Compound identification was achieved through comparison of retention times and UV spectral characteristics with authenticated reference standards. Catechin and procyanidin B2 tentatively identified as major constituents of the extract.

### 2.4. Animals and Ethical Considerations

Adult female CD-1 mice (8–10 weeks old, 25–30 g) were obtained from the animal facility of the Faculty of Medicine, National Autonomous University of Mexico (UNAM), Mexico City, Mexico. Animals were housed under standardized environmental conditions (23 ± 2 °C, 12 h light/dark cycle, 50–60% relative humidity) with unrestricted access to standard laboratory chow and water *ad libitum*. Prior to ethanol administration, animals were fasted for 24 h while maintaining free access to water. Following acclimatization, mice were randomly assigned to experimental groups to minimize selection bias and improve reproducibility. All experimental procedures complied with the Mexican Official Standard NOM-062-ZOO-1999 governing the care and use of laboratory animals and were conducted in accordance with the ARRIVE 2.0 guidelines for reporting animal research, ensuring transparency, reproducibility, and adherence to contemporary ethical standards. Experimental protocols were approved by the Institutional Committee for Animal Care and Use of the Faculty of Medicine, UNAM (Approval No. FM/DI/99/2025). At the conclusion of the study, animals were humanely euthanized under anesthesia prior to tissue collection.

### 2.5. Experimental Design and Treatment Protocol

This study was conducted in three sequential experimental phases: (a) standardization of the ethanol-induced gastric injury model: (b) evaluation of the dose–response relationship of the extract; and (c) assessment of the selected dose using macroscopic, histological, bio-chemical, and molecular endpoints. Animals were randomly assigned to experimental groups (*n* = 5–8 per group) to evaluate the gastroprotective effects of a polyphenol-rich hydroethanolic leaf extract of Pen against ethanol-induced acute gastric injury. A preliminary dose–response study was performed using oral doses of 5.5, 11, 16.5, and 22 mg/kg administered once daily for seven consecutive days. The extract was freshly suspended in distilled water immediately before administration. The dose range was established based on preliminary pharmacological observations and apparent tolerability of the extract. During preliminary experiments, the highest tested dose (22 mg/kg) showed no overt signs of poor tolerability or treatment-related deterioration in the general condition of the animals. Lower doses of 5.5, 11 and 16.5 mg/kg, corresponding to 25%, 50% and 75% of the highest dose, respectively, were subsequently evaluated. Among the doses tested, 22 mg/kg produced the greatest reduction in gastric lesion formation and was therefore selected for all subsequent analyses. The induction of experimental gastric injury was performed using the ethanol model described in Section 2.6. Briefly, animals received a single oral administration of 50% ethanol after the treatment period, and gastric tissues were collected one hour later for analysis. Famotidine (20 mg/kg, oral administration) was included as a positive control during model validation because of its well-established gastroprotective activity. Following confirmation of model responsiveness, the famotidine group was not included in the subsequent exploratory molecular analyses, in accordance with the reduction principle of the 3Rs framework for ethical animal experimentation. Famotidine was retained as a positive control for the macroscopic and histopathological, assessments used to compare the gastroprotective effects of Pen with the reference drug.

The experimental groups were as follows:

- Control (Ctrl): distilled water for seven consecutive days.
- Ethanol (EtOH 50%): distilled water for seven days followed by 50% ethanol administration.
- Pen (22 mg/kg): *M. bipinnatifida* extract re-suspended in distilled water for seven consecutive days.
- Pen + EtOH (22 mg/kg + EtOH 50%): extract re-suspended in distilled water for seven consecutive days followed by ethanol administration.
- Positive control (Famotidine + EtOH 50%): famotidine treatment re-suspended in distilled water prior to ethanol administration.

Following euthanasia, stomachs were harvested for macroscopic lesion scoring, histopathological evaluation, and biochemical assessment of oxidative stress-related biomarkers (Figure 1).

### 2.6. Ethanol-Induced Gastric Injury Model

The ethanol-induced gastric injury model was performed according to previously validated methodologies for the evaluation of gastroprotective agents [20]. Prior to the experiment, mice were fasted for 24 h with free access to water. For model standardization, two ethanol concentrations (50% and 100%, *v*/*v*, prepared in distilled water) were evaluated to determine reproducible conditions for acute gastric injury induction. Ethanol solutions were administered orally by gavage (20G, Model: 7910, Cadence Science, RI, USA) at a dose of 2 g/Kg per animal. All ethanol administrations were performed during the light phase, between 07:00 and 09:00 h, to minimize potential variability associated with circadian rhythms. One hour after ethanol administration, animals were euthanized by intraperitoneal overdose of sodium pentobarbital (150 mg/kg). Stomachs were immediately excised, opened along the greater curvature, and rinsed with physiological saline solution to remove gastric contents. Macroscopic examination was performed to evaluate hemorrhagic lesions and determine the gastric lesion area. Gastric tissues were subsequently processed for histopathological and biochemical analyses. Based on lesion severity and experimental reproducibility, 50% ethanol was selected for subsequent experiments.

### 2.7. Measurement and Quantitative Analysis of Gastric Lesion Area

Macroscopic images of gastric tissues were captured and quantitatively analyzed using ImageJ software (Version 1.53k, National Institutes of Health, Bethesda, MD, USA). Gastric lesions, including hemorrhagic and erosive areas, were delineated using a standardized color thresholding method and quantified relative to the total stomach area under identical analytical conditions for all experimental groups. The percentage of gastric mucosal injury was calculated using the following equation:

Gastric injury (%) = (Lesion area/Total stomach area) × 100

All macroscopic evaluations were performed in a blinded manner to minimize observer bias.

### 2.8. Histopathological Study

Histopathological assessment of gastric injury was performed using hematoxylin and eosin (H&E) staining following established protocols for ethanol-induced gastric injury [9]. Gastric tissues were fixed in 10% neutral-buffered formalin for 24 h, dehydrated through graded ethanol solutions, cleared in xylene, embedded in paraffin, and sectioned at 5 μm using a rotary microtome. Sections were mounted on glass slides, deparaffinized, rehydrated, stained with H&E, and permanently mounted using Entellan^®^ resin (Merck, Darmstadt, Germany). Histological examination was performed using a Nikon E200 light microscope (Nikon, Melville, NY, USA) at 10× and 40× magnifications. Digital images were acquired under identical illumination and acquisition settings for all experimental groups. For each animal, five randomly selected, non-overlapping microscopic fields from the glandular region of the stomach were analyzed.

Histopathological evaluation was conducted independently by two blinded observers. Gastric mucosal injury was assessed based on established morphological criteria, including epithelial integrity, mucosal erosion, glandular architecture, submucosal edema, hemorrhage, vascular congestion, and inflammatory cell infiltration. Representative photomicrographs were obtained for each experimental group. Morphometric analysis was performed using ImageJ software (Version 1.53k, National Institutes of Health, Bethesda, MD, USA). After image calibration, damaged mucosal areas were manually delineated and quantified as the percentage of injured tissue relative to the total mucosal area examined. The animal, rather than the individual microscopic field, was considered the experimental unit. The mean value obtained from the five analyzed fields for each animal was used for statistical analysis.

### 2.9. Assessment of Lipid Peroxidation by the TBARS Assay

Following euthanasia by sodium pentobarbital overdose, stomach tissues were excised, placed on a prechilled surface, and hemisected. Gastric tissue samples were rinsed with phosphate-buffered saline (PBS, 0.02 M, pH 7.2), weighed (25 mg), and homogenized in radioimmunoprecipitation assay (RIPA) buffer supplemented with a protease inhibitor cocktail. Homogenization was performed by sonication for 15 s at 40 Hz under ice-cold conditions. Samples were centrifuged at 1600× *g* for 10 min at 4 °C, and supernatants were collected and stored at −80 °C until analysis. Protein concentration was determined in 20 μL aliquots using a bicinchoninic acid (BCA) protein assay kit (Cat. No. 23225, Pierce Thermo Fisher Scientific, Waltham, MA, USA). Lipid peroxidation was evaluated by measuring malondialdehyde (MDA) levels using a Thiobarbituric Acid Reactive Substances (TBARS) assay kit (Cat. No. KA1381, Abnova, Taipei, Taiwan), according to the manufacturer’s instructions.

### 2.10. Western Blot

Western blot analyses were performed as exploratory molecular assessments using three independent biological samples per group included in the molecular analysis (*n* = 3). The analysis included the control, ethanol-treated, and Pen + EtOH groups. Famotidine was not included in the Western blot analysis because this group had been used primarily as a positive control during model validation and was not included in the subsequent exploratory molecular assessment, in accordance with the reduction principle of the 3Rs framework. Stomach tissues were homogenized to obtain 10% (*w*/*v*) homogenates using an ice-cold buffer containing 50 mM Tris–HCl (pH 7.4), 8.5% sucrose, 2 mM EDTA, 2 mM EGTA, and 10 mM β-mercaptoethanol, supplemented with protease and phosphatase inhibitor cocktails. Protein concentration was determined using a bicinchoninic acid (BCA) protein assay kit (Cat. No. 23225, Thermo Fisher Scientific, Waltham, MA, USA). Equal amounts of protein (10 μg per lane) were mixed with Laemmli sample buffer, denatured at 95 °C for 5 min, and separated by 10% or 15% SDS-polyacrylamide gel electrophoresis (SDS-PAGE), depending on the molecular weight of the target protein. Proteins were transferred onto 0.2 μm Immobilon-P PVDF membranes (Millipore, Bedford, MA, USA). Membranes were blocked with 1% bovine serum albumin (BSA) in TBS-T for 1 h at room temperature and incubated overnight at 4 °C with the following primary antibodies: anti-TNF-α (1:1000, sc-52746, Santa Cruz Biotechnology, Dallas, TX, USA), anti-IL-1β (1:1000, PA20B, Invitrogen, Waltham, MA, USA), anti-SOD2 (1:1000, PA530604, Invitrogen USA), anti-catalase (1:500, PA529183, Invitrogen USA), and anti-β-actin (1:1000, Mouse Hybridoma, Cinvestav-IPN). After three washes with TBS-T, membranes were incubated for 1 h at room temperature with horseradish peroxidase-conjugated secondary antibodies (Jackson Immuno Research Laboratories, West Grove, PA, USA) diluted 1:10,000 in blocking solution. Immunoreactive bands were visualized using Immobilon^®^ Chemiluminescent HRP High Sensitivity Substrate (Sigma-Aldrich, St. Louis, MO, USA) and detected using a UVP Chem Studio imaging system (Analytik Jena, Jena, Germany). Band intensities were quantified using Image Lab software version 6.1 (Bio-Rad Laboratories, Hercules, CA, USA). Protein expression levels were normalized to the corresponding β-actin signal and expressed as relative optical density ratios. Western blot analyses were performed using three independent biological replicates per group included in the molecular analyses (*n* = 3). Representative immunoblots and corresponding densitometric analyses are presented in Section 3.

### 2.11. Statistical Analysis

Data are presented as mean ± standard error of the mean (SEM). Statistical analyses were performed using GraphPad Prism software (Version 10.0; GraphPad Software, San Diego, CA, USA). Data normality and homogeneity of variances were assessed using the Shapiro–Wilk and Brown–Forsythe tests, respectively. Differences among experimental groups were analyzed using one-way analysis of variance (ANOVA) followed by Tukey’s multiple-comparisons post hoc test. For Western blot analyses, densitometric values were normalized to the corresponding β-actin signal before statistical evaluation. A *p* value < 0.05 was considered statistically significant. Unless otherwise indicated, *n* represents independent biological replicates for each experimental group.

## 3. Results

### 3.1. Qualitative HPLC-DAD Characterization of the Hydroethanolic Extract of Pen

To verify the phytochemical profile of the hydroethanolic extract used in the present study, a qualitative HPLC-DAD analysis was performed. A comprehensive phytochemical characterization of *Montanoa bipinnatifida* leaf extract, including compound identification based on chromatographic and spectroscopic analyses, together with its antioxidant evaluation, has been previously reported by our research group [16]. Therefore, the objective of the present analysis was limited to confirming the qualitative chromatographic fingerprint of the extract batch used in the biological experiments.

The HPLC-DAD chromatogram recorded at 270 nm showed a chromatographic profile with two clearly detected peaks at retention times of 9.71 and 12.76 min (Figure 2). Based on comparison of retention times and UV absorption spectra with authentic analytical standards, these peaks were identified as catechin and procyanidin B2, respectively, consistent with our previous phytochemical characterization [16]. Additional peaks detected at retention times below 9 min were also consistent with phenolic constituents previously reported in *M. bipinnatifida* leaf extracts [16]. The qualitative chromatographic profile obtained for the extract batch used in the present study was consistent with the previously reported phytochemical fingerprint, particularly with respect to the detection of catechin and procyanidin B2 at comparable retention times [16]. Because the present HPLC-DAD analysis was designed for qualitative batch verification, quantitative concentrations of individual phenolic compounds were not determined; therefore, direct quantitative batch-to-batch comparisons could not be performed. This analysis was performed as a qualitative identity assessment of the extract batch used for the in vivo experiments and should not be interpreted as a quantitative evaluation of compound abundance or batch-to-batch reproducibility.

### 3.2. Selection of the Ethanol-Induced Gastric Injury Model

To establish a reproducible experimental model of acute gastric injury, the macroscopic effects of 50% and 100% ethanol were compared (Figure 3). Gross examination of gastric tissues revealed marked concentration-dependent differences in mucosal damage. The control group exhibited smooth gastric mucosa with preserved morphology, characterized by a homogeneous pink appearance without visible hemorrhage, edema, erosions, or vascular congestion (Figure 3A). In contrast, administration of 50% ethanol produced evident gastric injury, including longitudinal hemorrhagic streaks, diffuse hyperemia, focal erosions, and moderate disruption of the mucosal surface (Figure 3B). These lesions are consistent with the characteristic features of acute ethanol-induced gastric injury while maintaining sufficient tissue integrity for subsequent histological and biochemical analyses. Exposure to 100% ethanol resulted in substantially more severe gastric damage (Figure 3C), characterized by extensive hemorrhage, widespread mucosal erosion, pronounced vascular congestion, and severe disruption of tissue architecture. The lesions were larger and more diffuse than those observed after administration of 50% ethanol, indicating a greater degree of mucosal destruction. Quantitative morphometric analysis confirmed the macroscopic observations (Figure 3D,E). Both ethanol concentrations significantly increased the gastric lesion area and the percentage of damaged mucosal area compared with the control group. Moreover, absolute ethanol produced significantly greater gastric damage than 50% ethanol, as demonstrated by higher lesion area values and a larger percentage of injured tissue. confirming a concentration-dependent effect of ethanol on gastric mucosal injury.

Although both ethanol concentrations successfully induced acute gastric lesions, the 50% ethanol model generated consistent and reproducible gastric injury while preserving adequate tissue architecture for subsequent histopathological, biochemical, and molecular analyses. In contrast, administration of absolute ethanol caused extensive tissue destruction that could interfere with the accurate evaluation of gastroprotective responses. Therefore, 50% ethanol was selected for all subsequent experiments investigating the gastroprotective effects of Pen.

### 3.3. Evaluation of the Gastroprotective Effect Across the Tested Dose Range and Selection of the Experimental Dose

A dose–response study was conducted to determine the optimal gastroprotective dose of the hydroethanolic extract of Pen using the ethanol-induced acute gastric injury model. Animals were pretreated with 5.5, 11, 16.5, or 22 mg/kg of the extract prior to the administration of 50% ethanol. Gastroprotective efficacy was subsequently assessed through complementary macroscopic, morphometric, and histopathological analyses (Figure 4).

Macroscopic examination demonstrated severe gastric mucosal injury in the ethanol-treated group, characterized by extensive longitudinal hemorrhagic lesions, diffuse hyperemia, and marked disruption of mucosal integrity (Figure 4B). In contrast, pretreatment with *M. bipinnatifida* markedly attenuated these lesions in a dose-dependent manner (Figure 4C–F). While the lowest dose (5.5 mg/kg) afforded only partial protection, increasing doses progressively preserved the gastric mucosa, with the 22 mg/kg group exhibiting a macroscopic appearance closely resembling that of the untreated control animals. These observations were corroborated by quantitative morphometric analysis (Figure 4G). Ethanol administration significantly increased both the gastric lesion area (mm^2^) and the percentage of damaged gastric mucosa compared with the control group (*p* < 0.0001). Pretreatment with Pen significantly reduced both parameters at all tested doses relative to the ethanol-treated group, demonstrating a clear dose-dependent gastroprotective response. The greatest reduction in lesion area and damaged mucosal surface was achieved with the 22 mg/kg dose, which exhibited the highest degree of macroscopic protection.

Histopathological evaluation further confirmed the protective effects of the extract (Figure 4I–N). Gastric tissue from the control group displayed normal histological architecture, characterized by an intact surface epithelium, well-organized gastric glands, preserved mucosal integrity, and the absence of inflammatory changes (Figure 4I). Conversely, ethanol exposure produced severe gastric injury, including extensive epithelial erosion, disruption of glandular architecture, edema of the lamina propria, vascular congestion, and prominent inflammatory cell infiltration (Figure 4J), consistent with acute ethanol-induced gastritis. Pretreatment with Pen markedly attenuated these histopathological alterations in a dose-dependent manner. Animals receiving 5.5 mg/kg exhibited partial preservation of epithelial integrity and glandular organization, although inflammatory infiltration remained evident (Figure 4K). Administration of 11 mg/kg (Figure 4L) resulted in a substantial improvement in tissue architecture, accompanied by reduced edema and inflammatory cell infiltration. At 16.5 mg/kg (Figure 4M), gastric histology was largely preserved, with well-defined glandular structures and minimal inflammatory changes. The highest dose (22 mg/kg; Figure 4N) produced the greatest histological protection, with gastric sections displaying nearly complete preservation of epithelial continuity, normal glandular organization, and only minimal residual inflammatory alterations, closely resembling the morphology observed in the control group.

The remarkable agreement among the macroscopic observations, morphometric measurements, and histopathological findings supports a dose-dependent gastroprotective effect under the experimental conditions evaluated. Because the 22 mg/kg dose consistently provided the highest level of protection across all evaluated parameters, it was selected for all subsequent experiments investigating oxidative stress, endogenous antioxidant defenses, inflammatory mediators, and the mechanisms underlying the gastroprotective activity of the extract.

### 3.4. Comparison of the Gastroprotective Effect of Pen Extract and Famotidine

Following identification of the optimal experimental dose (22 mg/kg), the gastroprotective efficacy of Pen was compared with that of famotidine using the ethanol-induced gastric injury model (Figure 5). Macroscopic examination demonstrated preserved gastric morphology in the control group, whereas ethanol administration produced extensive hemorrhagic lesions, diffuse hyperemia, and severe disruption of the gastric mucosal surface (Figure 5A,B). Pretreatment with Pen markedly reduced the extent of visible gastric lesions (Figure 5C), resulting in a macroscopic appearance comparable to that observed in the famotidine-treated group (Figure 5D). Quantitative morphometric analysis confirmed these observations (Figure 5I,J). Ethanol administration significantly increased both the gastric lesion area (mm^2^) and the percentage of damaged mucosal area compared with the control group (*p* < 0.05). Pretreatment with Pen significantly reduced both parameters relative to the ethanol-treated group. No statistically significant differences were detected between the extract-treated and famotidine-treated groups with respect to the evaluated parameters. Histopathological evaluation was consistent with the macroscopic and morphometric findings (Figure 5E–H). Gastric tissue from the control group exhibited preserved epithelial continuity, well-organized gastric glands, and normal mucosal architecture without evidence of inflammatory alterations (Figure 5E). In contrast, ethanol administration induced severe epithelial erosion, disruption of glandular architecture, submucosal edema, vascular congestion, hemorrhagic areas, and inflammatory cell infiltration (Figure 5F). Pretreatment with Pen markedly attenuated these histopathological alterations, preserving epithelial integrity and glandular organization while reducing edema and inflammatory infiltration (Figure 5G). Similar histological preservation was observed in the famotidine-treated group (Figure 5H), which displayed intact mucosal architecture with only minimal residual tissue alterations. The histopathological findings were consistent with the macroscopic observations and morphometric analysis (Figure 5). Histopathological scores were significantly lower in the Pen-treated group than in the ethanol group, indicating attenuation of ethanol-induced gastric injury. No statistically significant differences were detected between the Pen-treated and famotidine-treated groups under the experimental conditions evaluated.

### 3.5. Effect of Pen on Gastric Lipid Peroxidation Assessed by TBARS

Lipid peroxidation was evaluated by measuring gastric malondialdehyde (MDA) levels following ethanol-induced gastric injury (Figure 6). Administration of 50% ethanol resulted in a marked increase in MDA concentration compared with the control group, indicating enhanced lipid peroxidation in gastric tissue. Pretreatment with Pen (22 mg/kg) significantly reduced gastric MDA levels relative to the ethanol-treated group. A comparable reduction was observed in animals receiving famotidine, and no statistically significant differences were detected between the extract-treated and famotidine-treated groups. Although MDA levels in the Pen-treated group remained above those of untreated controls, pretreatment with the extract markedly attenuated ethanol-induced lipid peroxidation. Thus, Pen pretreatment significantly attenuated the ethanol-induced increase in gastric MDA/TBARS levels.

### 3.6. Effect of Pen on Antioxidant Proteins (SOD2 and Catalase) and Inflammatory Mediators (IL-1β and TNF-α)

To further investigate the molecular responses associated with the gastroprotective effect of Pen, the expression of antioxidant proteins and inflammatory mediators was analyzed by Western blot (Figure 7) in the control, ethanol-treated, and Pen + EtOH groups. The famotidine group was not included in this exploratory molecular analysis, as described in the Experimental Design and Treatment Protocol. Ethanol administration modified the expression profile of antioxidant enzymes relative to the control group (Figure 7A–C). Ethanol administration was associated with an increase in SOD2 protein expression relative to the control group; however, this difference did not reach statistical significance (Figure 7A,B). Catalase expression showed only modest variations among the experimental groups. Pretreatment with Pen was associated with a trend toward lower SOD2 expression compared with the ethanol-treated group, whereas catalase expression showed a trend toward higher levels; however, neither difference reached statistical significance.

More pronounced changes were observed for inflammatory mediators. Ethanol administration significantly increased IL-1β protein expression compared with the control group (Figure 7D). Pretreatment with Pen significantly reduced IL-1β expression relative to the ethanol-treated group, restoring protein levels toward those observed in control animals. In contrast, TNF-α exhibited a different expression profile (Figure 7E). Ethanol administration altered TNF-α expression relative to the control group, whereas pretreatment with Pen did not produce a significant reduction compared with the ethanol-treated animals. No statistically significant differences were detected between these two groups. Overall, Western blot analysis demonstrated that Pen differentially modulated proteins associated with antioxidant defense and inflammatory signaling during ethanol-induced gastric injury, with the most evident effect observed in the attenuation of IL-1β expression. Among the inflammatory markers analyzed, the clearest treatment-associated change was observed for IL-1β.

## 4. Discussion

Ethanol-induced gastric injury is a well-established experimental model characterized by oxidative stress, epithelial disruption, microvascular dysfunction, and activation of inflammatory pathways, and is widely used to investigate potential gastroprotective agents [1,2,21,22]. In the present study, the hydroethanolic extract of Montanoa bipinnatifida significantly attenuated acute ethanol-induced gastric injury in CD-1 mice, as demonstrated by reduced gastric lesion area, preservation of mucosal architecture, and decreased lipid peroxidation. These findings provide convergent macroscopic, histopathological, and biochemical evidence supporting the gastroprotective activity of the extract, while the observed changes in antioxidant and inflammatory markers provide exploratory insights into potential mechanisms underlying this effect.

The gastroprotective activity of *M. bipinnatifida* is consistent with the phytochemical composition of the hydroethanolic extract. As confirmed by the qualitative HPLC-DAD fingerprint obtained for the extract batch used in this study and previously characterized by our group, catechin and procyanidin B2 were identified among the predominant phenolic constituents [18]. These flavan-3-ols have been associated with antioxidant, anti-inflammatory, and cytoprotective effects and may influence redox-sensitive cellular responses and inflammatory signaling pathways [9,10,23,24]. Therefore, these constituents may contribute to the biological activity of the extract; however, their individual contribution to the gastroprotective response cannot be established from the present data.

Previous experimental evidence supports the potential relevance of catechin in gastric protection. Purified catechin has been reported to reduce ethanol-induced gastric ulceration and oxidative damage in experimental models [25]. This observation is consistent with the present findings, in which *M. bipinnatifida* pretreatment reduced gastric lesion formation and lipid peroxidation following ethanol exposure. Nevertheless, the presence of catechin in the extract does not establish that this compound is solely responsible for the observed gastroprotective effect. Procyanidin B2 may also contribute to the biological activity of the extract, although the available evidence is stronger for its antioxidant and anti-inflammatory properties than for its direct gastroprotective activity. Procyanidin B2 has been reported to modulate inflammatory signaling and reduce IL-1β production in experimental models of inflammation [26]. This finding is of particular interest in the context of the present study, in which *M. bipinnatifida* treatment significantly reduced gastric IL-1β expression following ethanol challenge.

Importantly, the hydroethanolic extract represents a chemically complex phytochemical matrix containing multiple phenolic and other bioactive constituents. Consequently, additive, or synergistic interactions among catechin, procyanidin B2, and other constituents may contribute to the overall biological response. Because the HPLC-DAD analysis performed in the present study was primarily qualitative, other phenolic or non-phenolic compounds that were not detected or structurally characterized may also contribute to the observed effects. Thus, the present findings support catechin and procyanidin B2 as plausible contributors to the gastroprotective activity of *M. bipinnatifida*, but the observed effect should be interpreted as the integrated biological activity of the extract rather than as the effect of a single constituent. Definitive determination of the contribution of individual compounds will require quantitative phytochemical characterization, isolation of individual constituents, and independent as well as combination experiments using purified compounds.

Oxidative stress is a central component of ethanol-induced gastric injury. Ethanol exposure can promote excessive reactive oxygen species (ROS) generation, resulting in lipid peroxidation, mitochondrial dysfunction, impairment of the mucosal barrier, and activation of inflammatory responses that contribute to epithelial damage [27,28,29]. Consistent with this mechanism, ethanol administration in the present study induced severe gastric lesions accompanied by a marked increase in gastric malondialdehyde (MDA), a commonly used marker of lipid peroxidation. Pretreatment with *M. bipinnatifida* significantly reduced gastric MDA levels, together with decreased gastric lesion area and preservation of mucosal histoarchitecture. Because lipid peroxidation can compromise membrane integrity and epithelial barrier function, the reduction in MDA is consistent with attenuation of oxidative injury as one component of the gastroprotective response. These findings are in agreement with previous studies identifying oxidative damage as an important contributor to ethanol-induced gastric injury [27,29].

Recent experimental studies further support the close relationship between oxidative stress and inflammatory responses in ethanol-induced gastric injury. Fan et al. [30] reported that *Dendrobium officinale* flos extract attenuated ethanol-induced acute gastric mucosal injury while reducing oxidative stress and inflammatory responses, including TNF-α and IL-6, and suppressing COX-2, iNOS, p38 MAPK, and NF-κB-related signaling. Similarly, Elbaz et al. [7] reported protective effects of dimethyl fumarate against ethanol-induced gastric ulcers through modulation of antioxidant and inflammatory pathways. More recently, Li et al. [31] showed that *Bletilla ochracea* extract protected against ethanol-induced acute gastric ulcers and was associated with activation of the Nrf2/HO-1/NQO1 antioxidant pathway and suppression of NF-κB p65/TLR4 signaling. These studies reinforce the relevance of redox and inflammatory processes in ethanol-induced gastric injury, although the specific molecular pathways involved may differ according to the intervention and experimental model. In this context, the significant reduction in gastric MDA levels observed following *M. bipinnatifida* treatment is consistent with current evidence supporting the importance of limiting oxidative damage as a component of gastroprotection. Importantly, however, the present study did not directly evaluate Nrf2, NF-κB, MAPK, or related signaling pathways; therefore, these pathways should be considered potential mechanisms for future investigation rather than established mechanisms of action of *M. bipinnatifida*.

In addition to reducing lipid peroxidation, *M. bipinnatifida* was associated with changes in endogenous antioxidant defense markers. Ethanol exposure was associated with a modest increase in SOD2 protein expression, whereas catalase showed a tendency toward lower expression; however, neither change reached statistical significance. Pretreatment with the extract was associated with a decrease in SOD2 expression relative to the ethanol-treated group and a tendency toward increased catalase expression, but neither change was statistically significant. Therefore, the present data do not provide conclusive evidence that *M. bipinnatifida* modifies the expression of these antioxidant enzymes under the experimental conditions evaluated. The decrease in SOD2 protein abundance following extract treatment should not be interpreted as normalization or as evidence of reduced antioxidant capacity. Rather, it may be compatible with a lower oxidative stimulus following extract treatment, particularly in view of the significant reduction in gastric MDA levels. Nevertheless, the present study does not establish a causal relationship between reduced lipid peroxidation and changes in SOD2 protein abundance. Moreover, because enzymatic activity was not directly measured, changes in protein expression cannot be interpreted as evidence of altered SOD2 or catalase activity. These findings should therefore be considered exploratory and interpreted in conjunction with the more robust evidence of reduced lipid peroxidation and preservation of gastric tissue architecture [1,32]. The relationship between oxidative stress and inflammation is particularly relevant to ethanol-induced gastric injury, as excessive ROS may contribute to the activation of inflammatory responses and amplification of mucosal damage. In the present study, ethanol administration significantly increased IL-1β expression, whereas pretreatment with *M. bipinnatifida* markedly reduced IL-1β expression [33]. The concomitant reduction in IL-1β, lipid peroxidation, and histopathological damage is consistent with a potential association between redox and inflammatory modulation and the gastroprotective response of the extract. However, the inflammatory response was not uniformly affected. TNF-α expression was increased following ethanol administration, and pretreatment with *M. bipinnatifida* did not suppress this response, with TNF-α remaining elevated in the Pen + EtOH group.

This differential cytokine response is important for the interpretation of the present findings. Although the extract clearly protected the gastric mucosa, the absence of TNF-α suppression indicates that its effects cannot be characterized simply as generalized anti-inflammatory activity. Instead, the findings suggest selective or differential modulation of inflammatory mediators under the experimental conditions evaluated. Such differential regulation is biologically plausible because plant-derived phenolic compounds can affect multiple inflammatory pathways, and their effects on individual cytokines may vary according to the experimental model, dose, treatment duration, and timing of evaluation [30,34]. Previous studies of plant-derived interventions in ethanol-induced gastric injury have similarly reported changes in inflammatory mediators, including TNF-α, IL-1β, and IL-6, together with modulation of NF-κB- and MAPK-related pathways [30,35]. However, because these pathways were not directly evaluated in the present study, they cannot be invoked as established mechanisms underlying the differential IL-1β and TNF-α responses observed here [36].

The elevated TNF-α expression in the Pen + EtOH group should also be interpreted cautiously. Cytokine responses are influenced by biological and experimental variability, and the Western blot analyses in the present study included a relatively limited number of biological replicates (*n* = 3 per group), reducing statistical power. Consequently, the observed TNF-α pattern requires independent replication in larger experimental groups before its biological significance can be established. In addition, cytokine expression was evaluated at a single experimental time point, and circulating TNF-α levels, TNF receptor activation, downstream signaling, and markers of epithelial repair or regeneration were not assessed. Therefore, the present data do not allow determination of whether the observed gastric TNF-α response represents a transient local response, a systemic inflammatory response, or a biologically relevant component of the protective phenotype. Future studies should incorporate temporal analyses and additional inflammatory and regulatory mediators, including IL-6 and IL-10, together with measurements of circulating cytokines and relevant signaling pathways [6,30,35,37]. These approaches will help determine whether the differential IL-1β and TNF-α responses represent reproducible biological effects and clarify their relevance to the gastroprotective activity of *M. bipinnatifida* [34].

The limited number of biological replicates in the Western blot analyses also warrants caution in interpreting the other molecular findings. Apparent trends, such as the tendency toward increased catalase expression following *M. bipinnatifida* treatment, should not be interpreted as definitive biological effects in the absence of statistical significance. Likewise, the absence of statistically significant differences should not be interpreted as evidence of equivalence or as definitive evidence that a biological effect is absent, particularly in the context of limited statistical power. Accordingly, the Western blot findings should be considered exploratory and hypothesis-generating. Larger, independently replicated studies will be necessary to determine whether the observed patterns are reproducible and whether they are mechanistically related to the gastroprotective phenotype. Future experiments should also assess antioxidant enzyme activities, glutathione homeostasis, and additional markers of oxidative damage to provide a more comprehensive evaluation of tissue redox status.

Regarding the reference treatment, no statistically significant differences were detected between the *M. bipinnatifida*-treated and famotidine-treated groups under the experimental conditions evaluated. However, this finding should not be interpreted as evidence of equivalent efficacy, because the present study was not designed or powered to establish pharmacodynamic equivalence or non-inferiority. Famotidine acts primarily through histamine H2-receptor blockade and suppression of gastric acid secretion, whereas the biological profile observed with *M. bipinnatifida* was associated with reduced lipid peroxidation and selective modulation of inflammatory markers. Thus, the two interventions may involve distinct biological processes, although the mechanisms responsible for the effects of the extract remain to be established experimentally. The comparison with famotidine therefore provides contextual information regarding the magnitude of the observed gastroprotective response rather than evidence of therapeutic equivalence.

Several limitations should be acknowledged. First, the study was conducted exclusively in female CD-1 mice using an acute ethanol-induced gastric injury model and a preventive treatment protocol. Therefore, the findings cannot be directly extrapolated to male animals, chronic gastric disorders, or human disease. Sex-related biological differences may influence oxidative stress, inflammatory responses, and responses to pharmacological or phytochemical interventions. Future studies including both sexes and, where appropriate, chronic, or therapeutic models will be important to determine the generalizability and translational relevance of the observed effects. Second, the relatively limited number of biological replicates in the Western blot analyses reduces statistical power, and these molecular findings should consequently be regarded as exploratory. Third, oxidative responses were evaluated primarily through MDA levels and antioxidant protein expression; enzymatic activity, glutathione homeostasis, and additional markers of oxidative damage were not assessed. Fourth, HPLC-DAD was used primarily for qualitative characterization of the extract batch, and quantitative concentrations, tissue exposure, pharmacokinetic behavior, and the individual contribution of catechin and procyanidin B2 were not determined. Finally, signaling pathways such as Nrf2/HO-1, NF-κB, MAPK, and NLRP3 were not directly evaluated, and therefore their involvement remains hypothetical.

From a translational perspective, the present findings provide a preclinical rationale for further investigation of *M. bipinnatifida* as a potential source of gastroprotective bioactive compounds but should not be interpreted as evidence of clinical efficacy. The acute ethanol-induced gastric injury model provides a controlled experimental system for evaluating mucosal damage and selected biochemical and inflammatory responses; however, it does not reproduce the full complexity of human gastric disease, including differences in chronicity, etiology, comorbidities, pharmacological exposure, and interindividual variability. Moreover, the present study was conducted exclusively in female CD-1 mice using an acute preventive protocol, which further limits direct extrapolation to human therapeutic use.

Translation toward clinical application would therefore require a stepwise development strategy. The gastroprotective effects should first be confirmed in larger, independently replicated studies and complementary models with greater translational relevance, including both sexes and, where appropriate, therapeutic rather than exclusively preventive treatment paradigms. In parallel, rigorous chemical standardization of the extract will be necessary, including quantitative characterization of its principal constituents and assessment of batch-to-batch consistency. Subsequent studies should establish its safety profile, pharmacokinetic and bioavailability characteristics, exposure–response relationship, appropriate dose range, and acute and repeated-dose toxicological profiles. The contribution of catechin and procyanidin B2 should also be investigated using purified compounds, individually and in combination, to determine their potential contribution to the observed effects. Only after reproducible efficacy, standardized composition, pharmacological exposure, and safety have been established would early-phase clinical studies be warranted to evaluate safety, tolerability, pharmacokinetics, and dose selection before efficacy is assessed in adequately controlled clinical trials.

The present findings also highlight several mechanistic questions that warrant further investigation. Future studies should complement protein-expression analyses with direct measurements of SOD2 and catalase enzymatic activities, together with assessment of the glutathione redox system, including GSH, GSSG, and the GSH/GSSG ratio. These complementary endpoints would help distinguish changes in protein abundance from functional enzymatic responses and provide a more comprehensive assessment of tissue redox homeostasis. In addition, temporal analyses, evaluation of inflammatory and signaling pathways, and assessment of epithelial repair and regeneration markers would help determine whether the differential TNF-α and IL-1β responses represent reproducible biological effects and clarify their relevance to the gastroprotective activity of *M. bipinnatifida*. Overall, the present findings support *M. bipinnatifida* as a candidate source of gastroprotective bioactive compounds and provide a basis for further studies aimed at establishing the reproducibility, chemical basis, safety, and mechanisms underlying its protective effects against acute ethanol-induced gastric injury.

## 5. Conclusions

The present study demonstrates that the hydroethanolic extract of *Montanoa bipinnatifida* protects against acute ethanol-induced gastric injury in CD-1 mice. Treatment significantly reduced gastric lesion severity, preserved mucosal architecture, decreased lipid peroxidation, and reduced IL-1β expression, supporting a gastroprotective effect associated with attenuation of oxidative damage and selective inflammatory modulation. Although no statistically significant changes were observed in SOD2 and catalase protein expression, the convergent macroscopic, histopathological, and biochemical findings support the protective effect of the extract. Qualitative HPLC-DAD analysis confirmed the presence of catechin and procyanidin B2 in the extract, consistent with previous phytochemical characterization. No statistically significant differences were detected between the extract- and famotidine-treated groups under the experimental conditions evaluated; however, this finding should not be interpreted as evidence of equivalent efficacy. Further studies are required to identify the bioactive compounds responsible for these effects and to elucidate the molecular mechanisms underlying the gastroprotective activity of *M. bipinnatifida.* Future investigations should complement protein-expression analyses with direct measurements of SOD2 and catalase enzymatic activities, together with assessment of glutathione redox status, including GSH, GSSG, and the GSH/GSSG ratio. Multi-time-point studies and adequately powered experimental groups will also be important to clarify the temporal dynamics and reproducibility of the observed antioxidant and inflammatory responses, particularly the differential responses of IL-1β and TNF-α. In addition, direct evaluation of relevant signaling pathways, including Nrf2/HO-1 and NF-κB, together with appropriate pathway-specific approaches, will be necessary to determine whether these mechanisms causally contribute to the gastroprotective effects observed. Further studies using both sexes, complementary experimental models, standardized extract composition, and appropriate pharmacokinetic, bioavailability, and toxicological assessments will be necessary to establish the reproducibility, safety, translational relevance, and therapeutic potential of *M. bipinnatifida*.

## Figures and Tables

**Figure 1:**
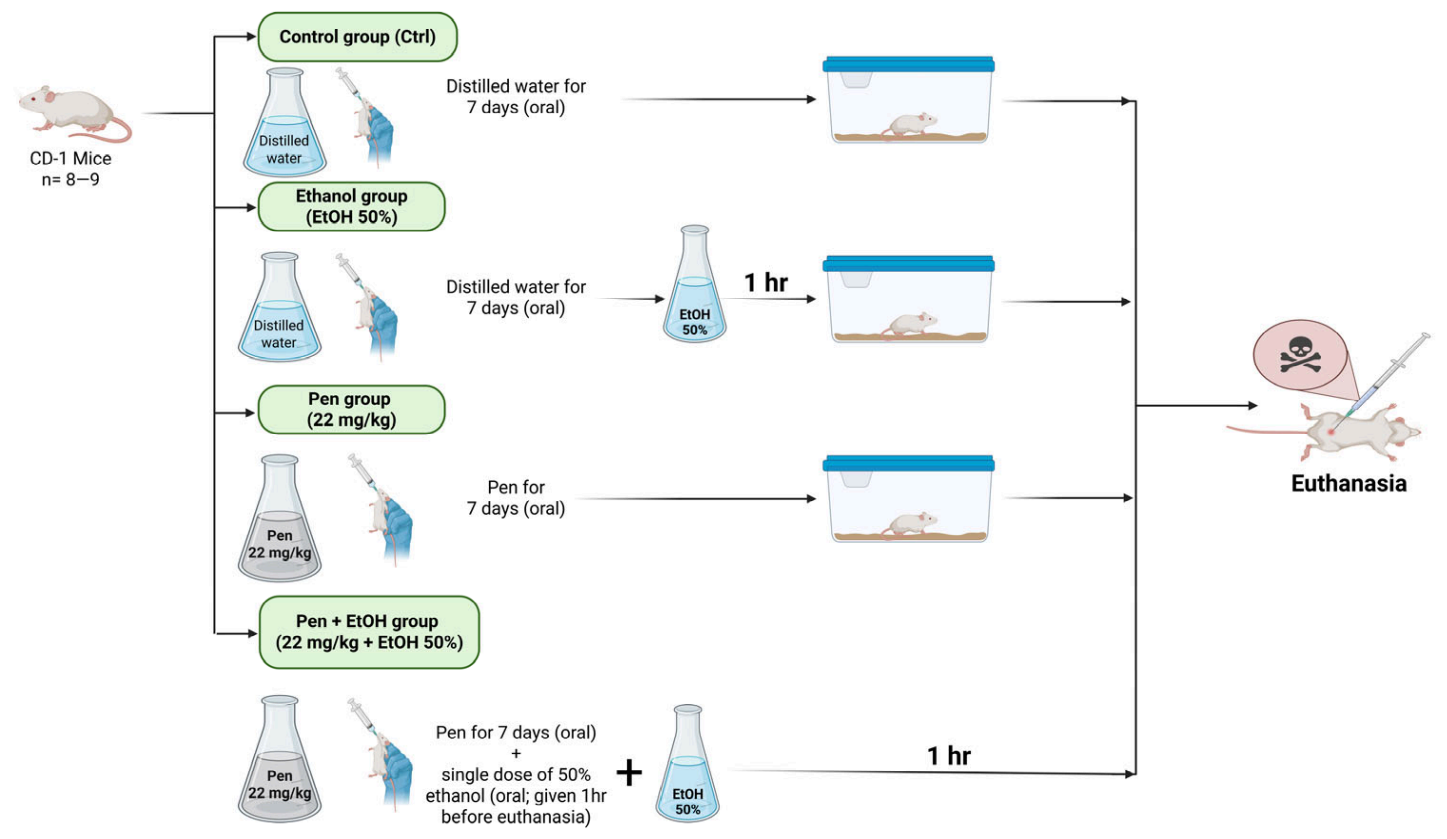
Schematic overview of the experimental protocol. Mice were treated orally with *Montanoa bipinnatifida* extract (Pen, 22 mg/kg) for seven days before ethanol challenge (50%, 0.2 mL/animal). Gastric tissues were collected one hour later for macroscopic, histopathological, and biochemical analyses. Created by BioRender.

**Figure 2:**
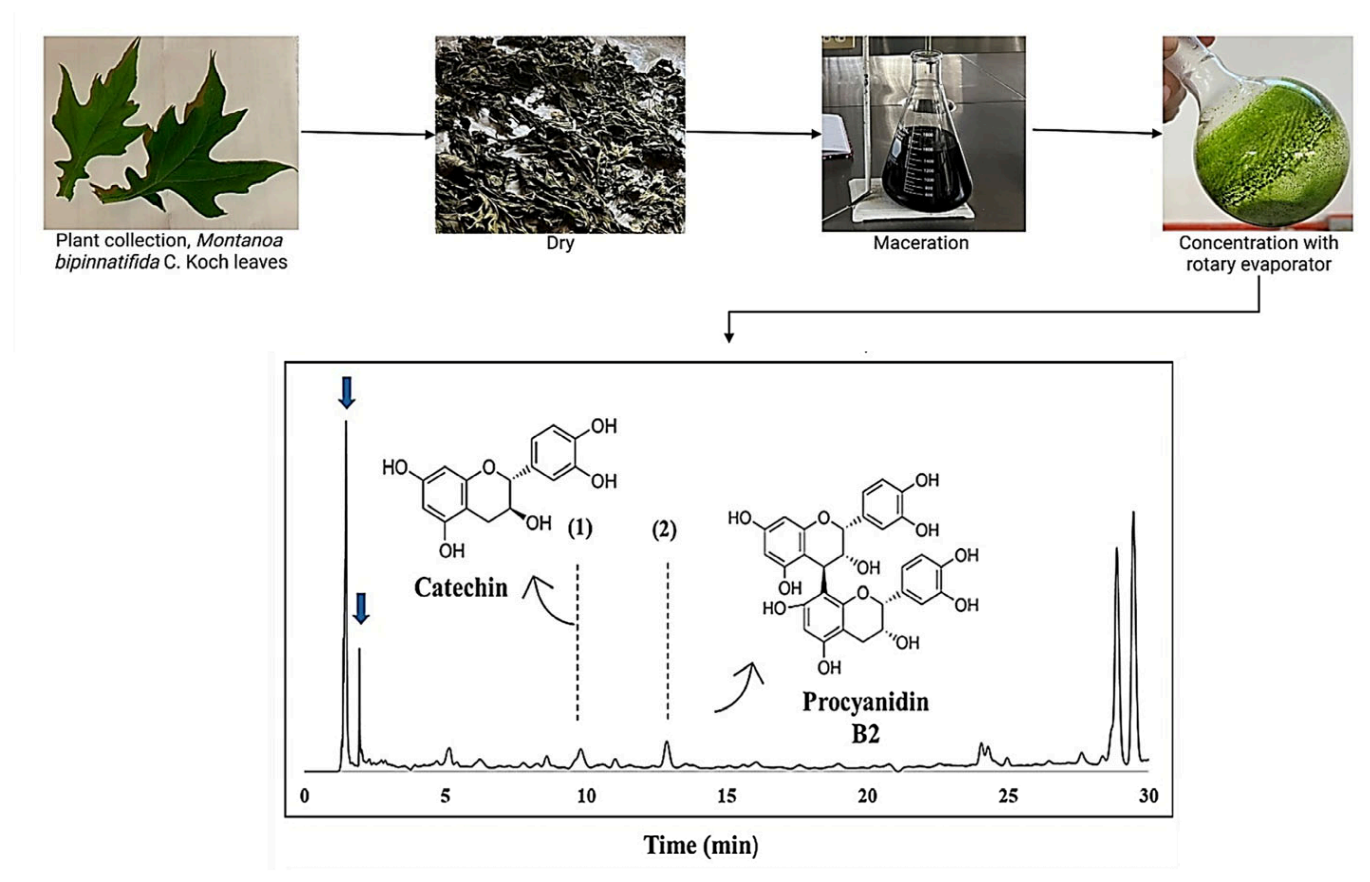
Preparation of the hydroethanolic extract of Pen leaves and qualitative HPLC-DAD chromatographic fingerprint. Fresh leaves were collected, dried, extracted by hydroethanolic maceration, and concentrated under reduced pressure. The chromatogram recorded at 270 nm shows the characteristic phenolic profile of the extract used in the present study. The major peaks correspond to (1) catechin (retention time: 9.71 min) and (2) procyanidin B2 (retention time: 12.76 min), identified by comparison with authentic analytical standards as previously reported by Gallegos-Hernández et al. [16]. The chromatographic analysis was performed as qualitative batch verification and was not intended for quantitative determination of individual phenolic compounds.

**Figure 3:**
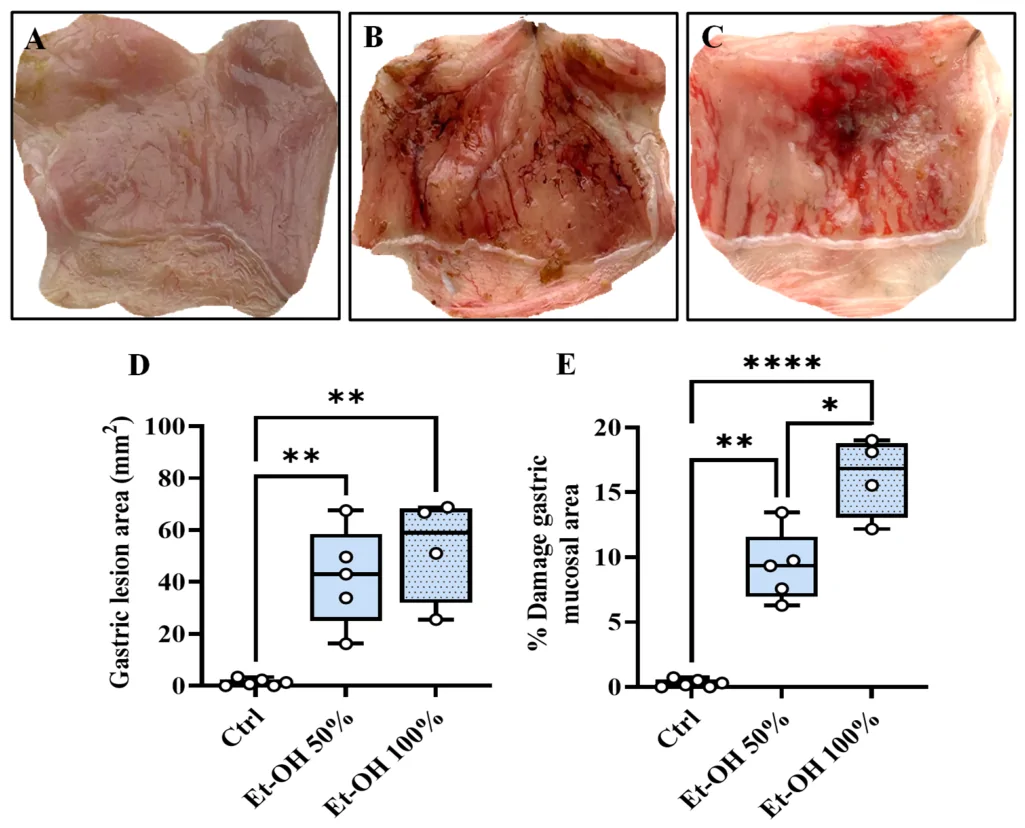
Selection of the experimental ethanol-induced gastric injury model. Representative macroscopic images of gastric mucosa following ethanol administration. (**A**) Control group showing normal gastric morphology with intact mucosal integrity. (**B**) Mice treated with 50% ethanol showing longitudinal hemorrhagic lesions, diffuse hyperemia, and focal mucosal erosions. (**C**) Mice treated with 100% ethanol showing extensive hemorrhage, widespread mucosal erosion, and severe disruption of gastric architecture. (**D**) Quantitative analysis of gastric lesion area (mm^2^), (**E**) Percentage of damaged gastric mucosal area. Data are presented as box-and-whisker plots, data are presented as mean ± standard error of the mean (SEM) (*n* = 4–6). Statistical significance is indicated as * *p* < 0.0193, ** *p* < 0.0022, and **** *p* < 0.001.

**Figure 4:**
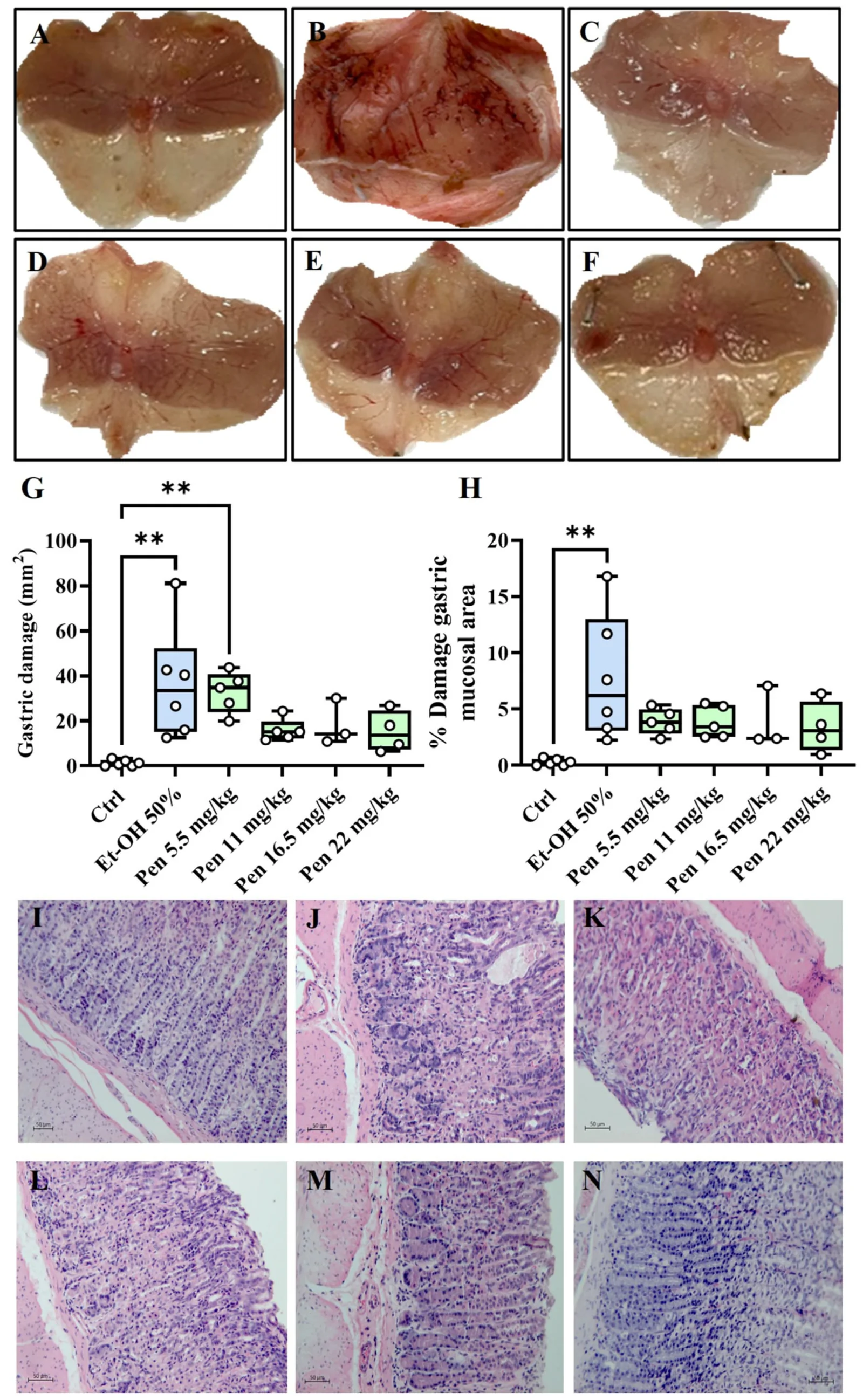
Dose-dependent gastroprotective effect of the hydroethanolic extract of *Montanoa bipinnatifida* against ethanol-induced gastric injury in CD-1 mice. Representative macroscopic appearance of gastric mucosa from (**A**) control animals, (**B**) ethanol-treated animals (50% EtOH), and animals pretreated with the extract at doses of (**C**) 5.5 mg/kg, (**D**) 11 mg/kg, (**E**) 16.5 mg/kg, and (**F**) 22 mg/kg prior to ethanol administration. Ethanol induced extensive hemorrhagic lesions and mucosal damage, whereas extract pretreatment progressively attenuated gastric injury in a dose-dependent manner. (**G**) Gastric lesion area (mm^2^) and (**H**) percentage of damaged gastric mucosal area. Data are presented as box-and-whisker plots, data are presented as mean ± standard error of the mean (SEM) (*n* = 4–6). Statistical analysis was performed using one-way ANOVA followed by Tukey’s post hoc test. ** *p* < 0.0001; ** *p* < 0.05. (**I**–**N**) Representative H&E-stained gastric sections. (**I**) Control group showing preserved epithelial continuity and normal glandular organization; (**J**) ethanol-treated group showing severe epithelial erosion, glandular disorganization, vascular congestion, inflammatory infiltration, and submucosal edema; (**K**–**M**) extract-treated groups (5.5, 11, and 16.5 mg/kg, respectively) showing partial preservation of mucosal architecture and progressive reduction in tissue damage; and (**N**) 22 mg/kg-treated group showing marked preservation of gastric histoarchitecture with minimal inflammatory alterations. Scale bars: 5 mm (**A**–**F**) and 50 µm (**I**–**N**). Pen: hydroethanolic extract of *Montanoa bipinnatifida*.

**Figure 5:**
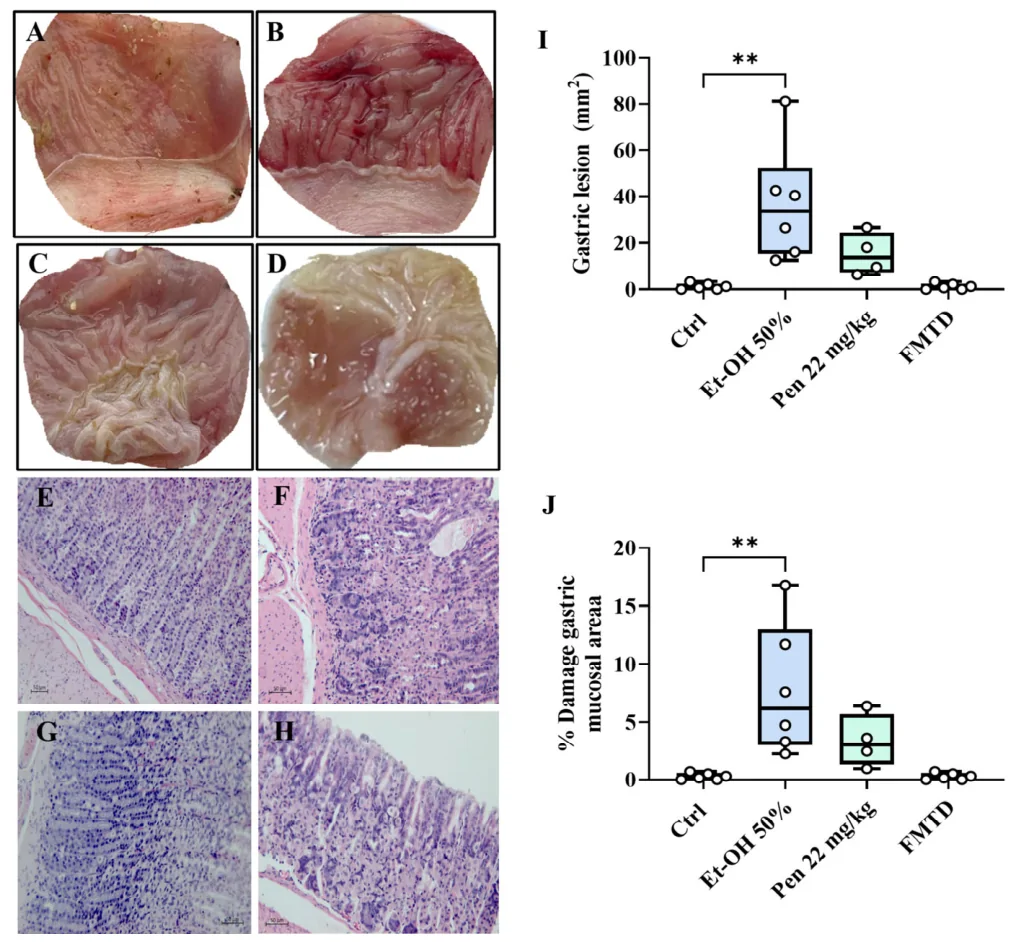
Comparison of the gastroprotective effects of Pen and famotidine in the ethanol-induced gastric injury model. Representative macroscopic images of gastric mucosa from (**A**) control, (**B**) ethanol-treated, (**C**) Pen (22 mg/kg), and (**D**) famotidine-treated animals. Representative hematoxylin and eosin (H&E)-stained gastric sections corresponding to (**E**) control, (**F**) ethanol-treated, (**G**) Pen, and (**H**) famotidine-treated groups. Control animals exhibited intact epithelial continuity and normal glandular architecture, whereas ethanol induced severe epithelial erosion, glandular disorganization, vascular congestion, submucosal edema, and inflammatory cell infiltration. Pretreatment with Pen markedly preserved gastric histoarchitecture and reduced inflammatory changes, showing a histological pattern comparable to that observed following famotidine treatment. (**I**) Quantification of gastric lesion area (mm^2^). (**J**) Percentage of damaged gastric mucosal area. Data are presented as box-and-whisker plots, data are presented as mean ± standard error of the mean (SEM) (*n* = 4–6). Statistical significance is indicated as ** *p* < 0.0008 (**I**) and ** *p* < 0.0015 (**J**). Scale bars: 5 mm (**A**–**D**) and 50 µm (**E**–**H**). Pen: hydroethanolic extract of *Montanoa bipinnatifida*.

**Figure 6:**
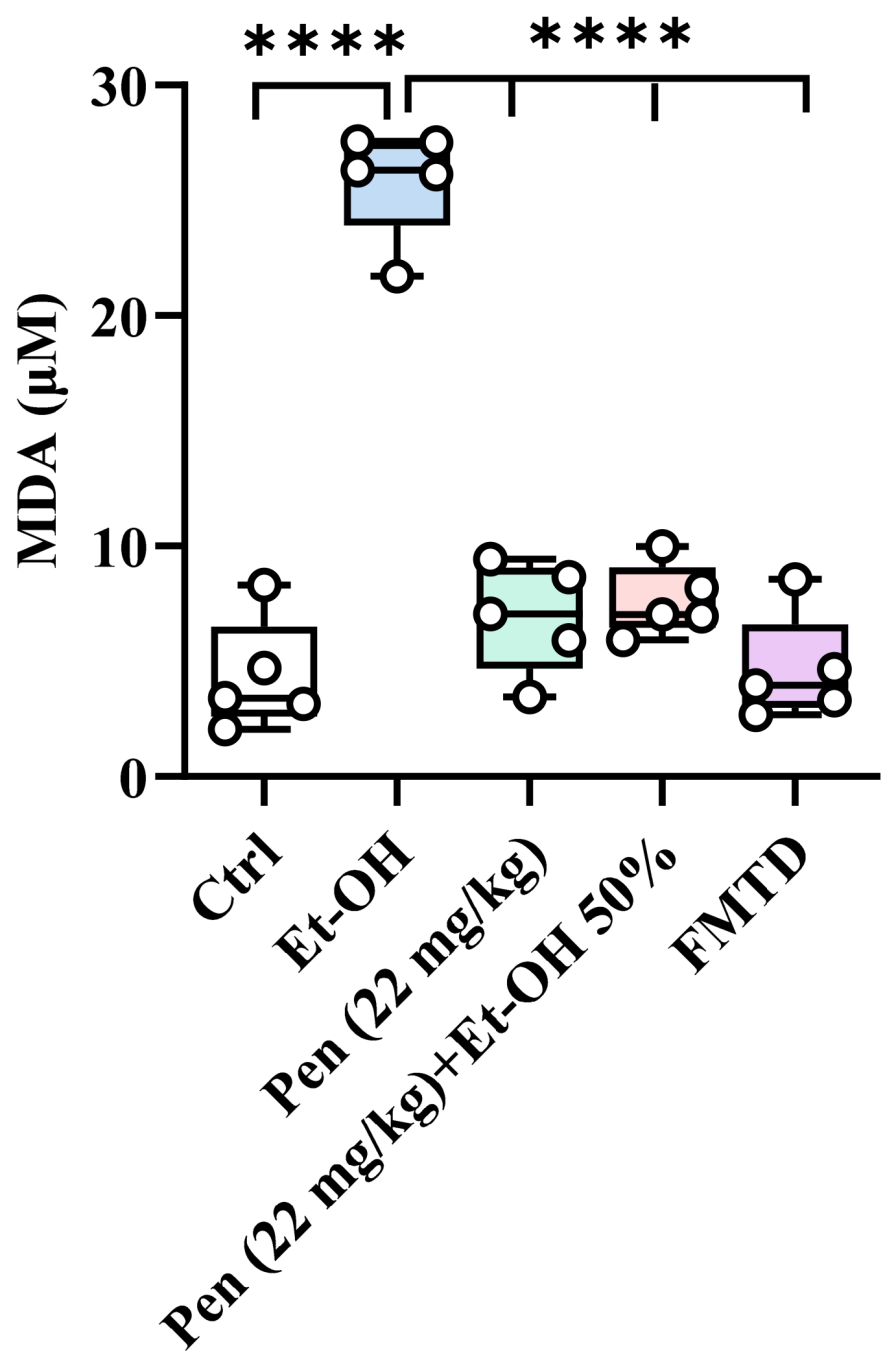
Effect of Pen hydroethanolic extract on gastric malondialdehyde (MDA) levels in the ethanol-induced gastric injury model. Gastric MDA concentrations were determined in control, ethanol-treated, Pen-treated (22 mg/kg), and famotidine-treated animals. Data are presented as box-and-whisker plots; data are presented as mean ± standard error of the mean (SEM) (*n* = 4–6). Statistical analysis was performed using one-way ANOVA followed by Tukey’s multiple-comparison test. **** *p* < 0.0036 versus the ethanol-treated group.

**Figure 7:**
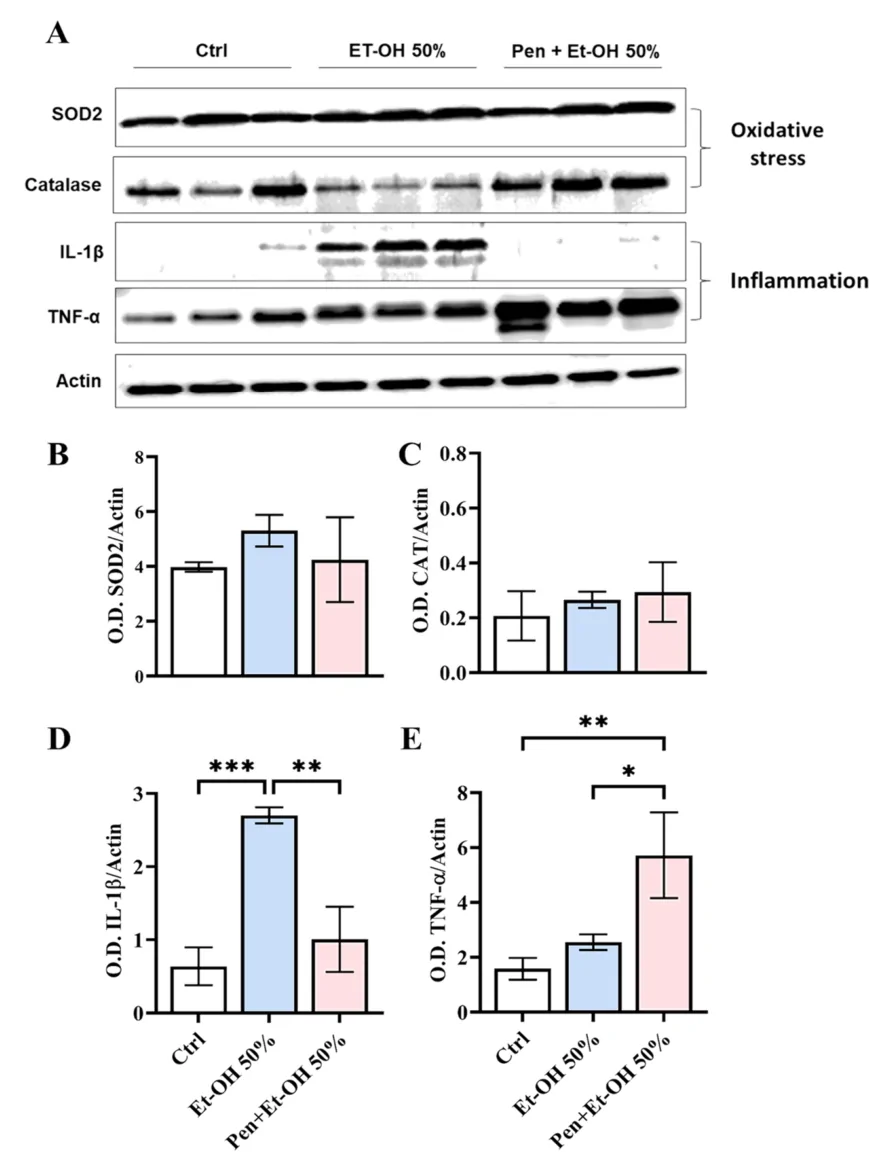
Effect of Pen hydroethanolic extract on the expression of antioxidant enzymes and inflammatory mediators in gastric tissue following ethanol-induced injury. (**A**) Representative Western blot images showing the protein expression of SOD2, catalase, IL-1β, and TNF-α in gastric tissue from the control, ethanol-treated (50% EtOH), and Pen-treated (22 mg/kg + EtOH) groups. The Western blot analysis was limited to the groups included in the exploratory molecular assessment. β-Actin was used as the loading control. (**B**–**E**) Densitometric quantification of (**B**) SOD2, (**C**) catalase, (**D**) IL-1β, and (**E**) TNF-α protein expression normalized to β-actin. Data are presented as box-and-whisker plots, data are presented as mean ± standard error of the mean (SEM) (*n* = 3). Statistical significance was determined by one-way ANOVA followed by Tukey’s multiple-comparison test. * *p* < 0.05, ** *p* < 0.01, *** *p* < 0.001.

## Data Availability

The original contributions presented in this study are included in the article. Further inquiries can be directed to the corresponding author.

## Abbreviations

The following abbreviations are used in this manuscript:

Et-OH	Ethanol
Fmtd	Famotidine
FTIR	Fourier transform infrared spectroscopy
H&E	Hematoxylin and eosin
HPLC-DAD	High-performance liquid chromatography with diode array detection
HO-1	Heme oxygenase-1
IL-1β	Interleukin-1 beta
MDA	Malondialdehyde
MPO	Myeloperoxidase
NF-κB	Nuclear factor kappa B
NQO1	NAD(P)H quinone oxidoreductase 1
Nrf2	Nuclear factor erythroid 2–related factor 2
PBS	Phosphate-buffered saline
RIPA	Radioimmunoprecipitation assay buffer
ROS	Reactive oxygen species
SDS-PAGE	Sodium dodecyl sulfate–polyacrylamide gel electrophoresis
SOD2	Superoxide dismutase 2
TBARS	Thiobarbituric acid reactive substances
TLC	Thin-layer chromatography
TNF-α	Tumor necrosis factor alpha
UV-Vis	Ultraviolet–visible spectroscopy
WB	Western blot
Ctrl	Control group
